# Accuracy of cervical cytology: comparison of diagnoses of 100 Pap smears read by four pathologists at three hospitals in Norway

**DOI:** 10.1186/s12907-017-0058-8

**Published:** 2017-08-29

**Authors:** Sveinung Wergeland Sørbye, Pål Suhrke, Berit Wallem Revå, Jannicke Berland, Ramona Johansen Maurseth, Khalid Al-Shibli

**Affiliations:** 1 Department of Clinical Pathology, University Hospital of North Norway, 9038 Tromsø, Norway; 2 Department of Pathology, Vestfold Hospital, Tønsberg, Norway; 3 Jannicke Berland, Stavanger University Hospital, Stavanger, Norway; 4 Nordlandssykehuset HF, Department of Pathology, Bodø, Norway

## Abstract

**Background:**

Cervical cancer can be prevented by early detection and treatment for precancerous lesions. Since 1995, there has been a national cervical cancer screening program in Norway, where women aged 25–69 years are recommended to take Pap smears every three years. There are 17 cytology laboratories covering a population of 5 million people. The detection rate of cervical abnormalities varies from laboratory to laboratory. We wanted to investigate the accuracy of cytology diagnoses by four different pathologists at three different hospitals in Norway.

**Methods:**

One hundred Pap smears (20 Normal, 20 ASC-US, 20 LSIL, 20 ASC-H and 20 HSIL) screened at UNN in 2015 were evaluated by four pathologists at three hospitals in Norway. All patients were followed up through December 2016. Histologically confirmed high-grade dysplasia (CIN2+) was considered as study endpoint.

**Results:**

The number of Pap smears evaluated as abnormal (ASC-US+) by the four pathologists varied from 61 to 85. The number of high-grade cytology (ASC-H+) varied from 26 to 50. There was moderate agreement (weighted kappa 0.45–0.58) between the observers. There were 32 women with high-grade histology (CIN2+) in the follow-up, including 19 CIN2, 12 CIN3 and one squamous cell carcinoma (SCC). Using high-grade cytology (ASC-H+) as cut-off, the sensitivity for CIN2+ varied from 68.8% to 93.8% (mean 77.4%) and specificity from 70.6% to 95.6% (mean 81.3%). The pathologist with the highest sensitivity for CIN2+ had the highest false positive rate and the lowest specificity (*p*<0.05). The accuracy for CIN2+ varied from 74.1% to 83.8% (mean 79.4%). The Pap smear from the woman with cervical cancer was diagnosed as high-grade (ASC-H+) by one of the four pathologists.

**Conclusions:**

Cervical cancer screening based on cytology has limited accuracy. The study revealed a moderate agreement between the observers, along with a trade-off between sensitivity and specificity. This might indicate that hospitals with high detection rates of cervical cytology have higher sensitivity for CIN2+ but lower specificity.

**Electronic supplementary material:**

The online version of this article (10.1186/s12907-017-0058-8) contains supplementary material, which is available to authorized users.

## Background

Cervical cancer is caused by human papillomavirus (HPV) and develops over many years through a series of precancerous steps [1, 2]. The disease can be prevented by using the HPV vaccine or by screening with HPV test or Pap smears [3, 4]. Since 2009, there has been a HPV vaccination program for 12-year-old girls in Norway. The program’s coverage is around 80% [5]. Since November 2016, there has been an ongoing two-year catch-up vaccination program for 20–25 years old women where the expected coverage rate is 40–45% (www.fhi.no). Since 2015, there has been a pilot for HPV testing in primary screening in four counties [6]. In this pilot, women 34 years and older are randomized to Pap smear every three years or HPV test every five years [6]. However, in most parts of Norway, the cervical screening program is still based on cervical cytology [5].

Since 1995, there has been a national cervical cancer screening program in Norway, where women aged 25–69 years are recommended to take Pap smears every three years [5]. Women with high-grade cytology (ASC-H / HSIL) are referred to a gynecologist for colposcopy and biopsy. HPV test is used in triage of women with low-grade cytology (ASC-US / LSIL). The cervical screening program has a coverage of 60% after 3.5 years. The Norwegian Cancer Registry sends a reminder to women without a Pap smear after three years and a new reminder after four years. The coverage is 80% after 5 years [5]. Most Pap smears are taken by GPs, while some samples are taken by gynecologists. There are 17 different laboratories involved in the screening program, and most of these use liquid-based cytology (ThinPrep or SurePath).

It is well known that cervical cytology has limited sensitivity and reproducibility [7–12]. Diagnoses may vary from cytotechnician to cytotechnician, from pathologist to pathologist and from lab to lab [9, 11, 12]. All cervical cytology diagnoses, results of HPV tests and biopsies from all laboratories in Norway are reported to the Norwegian Cancer Registry, which drafts annual reports with feedback to each laboratory, including the distribution of their diagnoses compared with the national average [5] (Table 1).

**Table 1:** Distribution (%) of selected cytological diagnoses in different labs in Norway in 2015

Lab	Normal	ASC-US	LSIL	ASC-H	HSIL
OUS	86.9	5.0	0.9	1.0	1.1
Lab for pat/Furst	92.6^2^	3.0^3^	0.7	0.5	0.6^3^
HUS^1^	83.6	5.0	2.4	0.8	1.4^2^
St.Olav^1^	84.0	8.0^2^	1.1	1.8^2^	1.1
Molde	86.6	2.9	1.0	0.4	1.1
Gyn lab/unilabs	89.9	4.9	0.8	0.7	0.6^3^
Østfold	87.0	4.6	0.9	0.9	0.8
UNN	79.2^3^	4.8	4.6^2^	1.4^2^	1.1
Telemark	88.7	3.2	2.3	0.3	1.0
Innlandet,Lillehammer	93.0^2^	3.4	0.9	0.6	0.6
Vestre Viken	91.1	3.6	1.8	0.6	0.9
Ålesund	91.4^2^	5.1	1.4	0.5	0.6^3^
Nordland	86.4	3.6	1.9	1.1	0.8
SUS^1^	84.6	4.9	2.5	0.6	1.3^2^
Sørlandet	89.1	3.0	1.1	0.7	0.8
AHUS	93.4^2^	2.5	1.5	0.8	1.0
Vestfold	83.0	8.5^2^	2.1	2.0^2^	0.9
Total	88.1	4.3	1.6	0.9	0.9

Adapted from the Norwegian Cancer Screening Programme – annual report 2015 [5]

^1^Laboratories included in HPV primary screening pilot where women 34 years and older are randomized to Pap smear every three years or HPV test every five years

^2^Significantly higher than the average for Norway (*p*<0.05)

^3^Significantly lower than the average for Norway (*p*<0.05)

There is a high variability in detection rates across hospitals. This may be due to higher sensitivity, lower specificity, differences in HPV prevalence, cervical dysplasia and cancer in some parts of the country compared to other parts of the country, or a combination of these causes. We wanted to investigate the accuracy of cytology diagnoses by four different pathologists at three different hospitals in Norway.

## Methods

One hundred cervical cytological samples screened at UNN in 2015 with the diagnoses normal, ASC-US, LSIL, ASC-H and HSIL were sent to the Departments of Pathology in Bergen (HUS), Bodø (Nordland), Fredrikstad (Østfold), Stavanger (SUS) and Tønsberg (Vestfold). The pathologist at the Department of Pathology in Bergen did not have time to participate in the study, and he forwarded the slides to Stavanger without looking at them. Two cytotechnologist at the Department of Pathology in Fredrikstad diagnosed the slides, but they were trained to screen SurePath samples. Their results were therefore excluded from this study based on ThinPrep samples.

All slides were first screened by a cytotechnologist at UNN and then evaluated by a pathologist at UNN (P1, reference). The abnormal cells were marked on the slides before being dispatched for the study. The slides were not screened at the other hospitals. The four other pathologists (P2–P5) at other hospitals were to only evaluate the abnormal cells marked on the slides. The other pathologists were blinded for age, previous findings, clinical information and HPV result. Diagnoses from each of the four pathologists were compared with diagnoses from the three other pathologists. Women with abnormal findings at UNN were followed up according to national guidelines. In Norway, the Bethesda System for Reporting Cervical Cytology is used by all laboratories. All patients were followed up through December 2016. Histologically confirmed high-grade dysplasia (CIN2+) was considered as study endpoint (gold standard). When calculating the sensitivity and specificity, women with normal Pap smears, and women with low-grade cytology (ASC-US / LSIL) and negative HPV test without histology, were considered free of high-grade dysplasia (CIN1-).

All analyses were done in IBM SPSS Statistics, version 23, with Chi-square test for categorical variables and *t*-test for continuous variables. For accuracy of cytological diagnoses between different observers, we used weighted kappa with linear weights.

## Results

Of the 100 cervical cytology samples, 20 were diagnosed Normal, 20 ASC-US, 20 LSIL, 20 ASC-H and 20 HSIL at UNN. There were 32 women with high-grade histology (CIN2+) in the follow-up, including 19 CIN2, 12 CIN3 and one squamous cell carcinoma (SCC). There were no CIN2+ in women with Normal diagnosis, one CIN2+ in women with ASC-US diagnoses, three women with CIN2+ in the LSIL group, 10 CIN2+ in the ASC-H group and 18 CIN2+ in the HSIL group (Table 2). Using high-grade cytology (ASC-H+) as cut-off, the sensitivity for CIN2+ at UNN was 87.5% (28/32).

**Table 2:** Cytology diagnoses at UNN with HPV tests and biopsies

Diagnoses	Samples	HPV test	HPV pos	HPV pos (%)	Biopsy	CIN2+	PPV (%)
Normal	20	1	0	0.0	0	0	0.0
ASC-US	20	19	8	42.1	6	1	5.0
LSIL	20	19	15	78.9	17	3	15.0
ASC-H	20	17	16	94.1	20	10	50.0
HSIL	20	14	13	92.9	20	18	90.0
Total	100	70	52	74.3	63	32	32.0

*ASC-US* atypical squamous cells of undetermined significance, *LSIL* low-grade squamous intraepithelial lesion, *ASC-H* atypical squamous cells cannot exclude HSIL, *HSIL* high-grade squamous intraepithelial lesion, CIN2+ = CIN2, CIN3 and cancer, *PPV* positive predictive value

The number of samples diagnosed as “Normal” varied from 15 to 39 by the four pathologists, with a mean of 28.8. One pathologist (P2) had significantly fewer “Normal” cases than the average of the four pathologists (*p*<0.05) (Table 3). The corresponding variation of ASC-US, LSIL, ASC-H and HSIL were 17 to 24 (mean 19.8), 9 to 20 (mean 14.0), 10 to 18 (mean 13.3) and 16 to 32 (mean 24.0), respectively (Table 3), none of which were significant. There was moderate agreement between the observers (weighted kappa 0.45–0.58) (Table 4). The kappa statistics were not statistically different.

**Table 3:** Distribution of diagnoses per pathologist

Observer	Normal	ASC-US	LSIL	ASC-H	HSIL	Total
P1 (ref)	20	20	20	20	20	100
P2	15^1^	19	16	18	32	100
P3	23	19	20	14	24	100
P4	39	24	11	10	16	100
P5	38	17	9	11	25	100
Mean (P2–P5)	28.8	19.8	14.0	13.3	24.0	100.0

*ASC-US* atypical squamous cells of undetermined significance, *LSIL* low-grade squamous intraepithelial lesion, *ASC-H* atypical squamous cells cannot exclude HSIL, *HSIL* high-grade squamous intraepithelial lesion

^1^Significantly lower than the average for P2–P5 (*p*<0.05)

**Table 4:** Agreement between observers (weighted kappa)

Observer	P2	P3	P4	P5
P2	-	0.53	0.48	0.45
P3	0.53	-	0.50	0.49
P4	0.48	0.50	-	0.58
P5	0.45	0.49	0.58	-

< 0.00 = No agreement

0.00–0.20 = Slight agreement

0.21–0.40 = Fair agreement

0.41–0.60 = Moderate agreement

0.61–0.80 = Substantial agreement

0.81–1.00 = Almost perfect agreement

The agreement of the different diagnoses was higher for “Normal” and “HSIL” samples than the other diagnoses (ASC-US, LSIL and ASC-H) (Additional file 1: Tables S1–S5). The number for high-grade cytology (ASC-H+) varied from 26 (P4) to 50 (P2). Of 61 women with at least one high-grade cytology, 17 samples (27.9%) were considered high-grade by all four observers (Additional file 1: Figure S1). The number of true positive (CIN2+) using ASC-H+ as a cut-off varied from 22 to 30 (mean 24.8) (Additional file 1: Figure S2 and Table 5). The corresponding sensitivity for CIN2+ varied from 68.8% to 93.8% (mean 77.4%). One pathologist (P2) had significantly higher sensitivity than the average of the four pathologists (*p*<0.05) (Table 5). Of 32 women with CIN2+, 15 samples (46.9%) were considered high-grade by all four observers (Additional file 1: Figure S2). One woman with CIN2 was not considered to have high-grade cytology by any of the four observers (patient 57, Additional file 1: Table S3). The number of true negative (CIN1-) using LSIL- as a cut-off varied from 48 to 65 (mean 55.3). The corresponding specificity ranged from 70.6% to 95.6% (mean 81.3%) (Table 5). One pathologist (P2) had significantly lower specificity and one pathologist (P4) had significantly higher specificity than the average of the four pathologists (*p*<0.05) (see Table 5). The pathologist (P2) with the highest sensitivity for CIN2+ had the highest false positive rate and the lowest specificity (Table 5). The accuracy for CIN2+ varied from 74.1% to 83.8% (mean 79.4%). There were no statistically significant differences in accuracy (Table 5). The Pap smear from the woman with cervical cancer (SCC) was diagnosed as high-grade (ASC-H+) by one of the four pathologists (P2), while three pathologists diagnosed her as ASC-US (Additional file 1: Table S5). The woman had a positive HPV test for HPV type 16 (data not shown).

**Table 5:** True positive, true negative, sensitivity and specificity for CIN2+ per pathologists using ASC-H+ as cut-off

Observer	TP	TN	FP	FN	SE (%)	SP (%)	AU (%)	PPV (%)	NPV (%)
P1 (ref)	28	56	12	4	87.5	82.4	85.0	70.0	93.3
P2	30	48	20	2	93.8^1^	70.6^2^	82.2	60.0	96.0^1^
P3	24	54	14	8	75.0	79.4	77.2	63.2	87.1
P4	23	65	3	9	71.9	95.6^1^	83.8	88.5^1^	87.8
P5	22	54	14	10	68.8	79.4	74.1	61.1	84.4
Mean (P2–P5)	24.8	55.3	12.8	7.3	77.4	81.3	79.4	68.2	88.8

*TP* true positive, *TN* true negative, *FP* false positive, *FN* false negative, *SE* sensitivity, *SP* specificity, AU = AUROC = accuracy = (SE+SP)/2, *PPV* positive predictive value, *NPV* negative predictive value, ASC-H+ = ASC-H and HSIL, CIN2+ = CIN2, CIN3 and cancer

^1^Significantly higher than the average for P2–P5 (*p*<0.05)

^2^Significantly lower than the average for P2–P5 (*p*<0.05)

## Discussion

The study’s purpose was to investigate the accuracy of cytology diagnoses by four different pathologists at three hospitals using 100 Pap smears with different cytological diagnoses screened at UNN. The agreement of the cytological diagnoses between the four pathologists in this study was “moderate.” A moderate agreement is better than “fair,” but worse than “substantial.” The kappa statistics were not statistically different.

In Norway there are 17 cytology laboratories covering a population of 5 million people [5]. All the laboratories receive most of their samples from general practitioners in primary screening. The population in Norway is quite homogenous, where Norwegian women in the different parts of Norway are mostly the same. The differences between the various laboratories are probably caused by different interpretation of the Bethesda criteria. Two pathologists (P4 and P5) were from the same laboratory but still used very different diagnoses for the same patients.

In the ATHENA study, the sensitivity of cytology varied from 42.0% to 73.0% [12]. In our study, the sensitivity for CIN2+ varied from 68.8% to 93.8%, but all the smears were first screened at the same hospital, and abnormal cells were marked on the slide. It is easy to find abnormal cells on a slide full of marks. In a population with a given prevalence of CIN2+, the sensitivity of cytology is dependent on the detection rate. In the ATHENA study, the positivity rate of cytology in primary screening varied from 3.8% to 9.9% while the detection rate of HPV DNA test (Cobas 4800) varied from 10.9% to 13.4% [12]. In our study, the detection rate of high-grade cytology (ASC-H / HSIL) varied from 26.0% to 50.0%, while the detection rate of HPV DNA test (Cobas 4800) was 74.3% (52/70).

In our study, the accuracy varied from 74.1% to 83.8% (mean 79.4%). In five published studies the accuracy varied from 64.2% to 78.4% (mean 76.1%) (Table 6). There was less variation between the four pathologists in our study than between the five published studies. The mean accuracy of the four pathologists in our study was significantly higher than the mean of the five published studies (79.4% vs 76.1%, *p*<0.05).

**Table 6:** True positive, true negative, sensitivity and specificity for CIN2+ in different studies using ASC-US+ as cut-off

Study	TP	TN	FP	FN	SE (%)	SP (%)	AU (%)	PPV (%)	NPV (%)
Katki 2011 [10]	1 226	318 093	11 415	1 084	53.1^2^	96.5^1^	78.4^1^	9.7^2^	99.7^1^
Dillner 2008 [15]	242	22 883	1 031	139	63.5^1^	95.7^2^	79.6^1^	19.0^1^	99.4^2^
Castle 2012 [16]	136	18 190	926	260	34.3^2^	95.2^2^	64.7^2^	12.8^1^	98.6^1^
Szarewski 2008 [17]	256	236	444	17	93.8^1^	34.7^2^	64.2^2^	36.6^1^	93.3^2^
Sørbye 2011 [18]	48	92	77	8	85.7^1^	54.4^2^	70.1^2^	38.4^1^	92.0^2^
Total	1 908	359 494	13 893	1 508	55.9	96.3	76.1	12.1	99.6

*TP* true positive, *TN* true negative, *FP* false positive, *FN* false negative, *SE* sensitivity, *SP* specificity, AU = AUROC = accuracy = (SE+SP)/2, *PPV* positive predictive value, *NPV* negative predictive value, ASC-US+ = ASC-US, LSIL, ASC-H and HSIL, CIN2+ = CIN2, CIN3 and cancer

^1^Significantly higher than the average (*p*<0.05)

^2^Significantly lower than the average (*p*<0.05)

There is a trade-off between sensitivity and specificity in cervical cancer screening. In our study the pathologist with the significantly highest sensitivity for CIN2+ had the significantly lowest specificity. In general, laboratories with a high detection rate of cytology also have higher sensitivity for CIN2+. If the sensitivity is higher, the hospital detects more women with CIN2/3 that can be treated, and fewer women develop cervical cancer before the next screening round. When women with low-grade cytology (ASC-US / LSIL) are triaged with HPV test, a high detection rate of low-grade cytology should not be considered as a major problem. A false positive ASC-US will have a negative HPV test and does not need follow-up. A false negative “Normal” cytology has no indication for HPV testing, according to Norwegian guidelines (www.kreftregisteret.no).

Cytology is subjective with poorly reproducible criteria. HPV testing is more objective with strictly defined criteria. Co-testing with both cytology and HPV test may reduce the risk of false negative cytology when the pathologists take the HPV result in consideration when evaluating the cytological slide. In our study, only the observer at UNN (P1, reference) knew the HPV result. All other observers were blinded for clinical information and HPV result, which might explain the lower sensitivity for CIN2+ for some of the other pathologist. Originally, in the ATHENA study, cytology was reviewed blinded to HPV status. When the same slides were re-reviewed unblinded to HPV status, the sensitivity for CIN3+ of co-testing increased from 54.1% to 62.4% (*P* = 0.0015) [13]. In our study, the mean sensitivity for CIN2+ for the four external pathologists was 77.4% based on slides screened at the same hospital.

The present study also has other weaknesses. For P1 the diagnoses were set in normal routine work, while the cases for the other four pathologists had to be diagnosed in addition to normal workload. This might affect the interpretation. In addition, only P1 at UNN had access to the initial diagnoses suggested by the cytotechnician. In daily practice the pathologist usually compares his or her initial impression with the diagnosis suggested by the cytotechnician. If there is discrepancy, the slide is reviewed. This might explain the lower sensitivity of some of the other pathologist. In normal routine work, difficult cases will be discussed with other pathologists. In this study, the pathologists reviewed all the slides alone.

Out of the 100 women in this study, there was one woman with cervical cancer. Three of the four pathologists diagnosed her cytology as ASC-US. According to Norwegian guidelines, women with ASC-US and a positive HPV result should be followed up with a new cytology and HPV test after 6–12 months. Only women with persistent HPV infection should be referred to a gynecologist for colposcopy and biopsy (www.kreftregisteret.no). This may delay diagnosis, treatment and worsen her prognosis.

There were statistically significant differences in sensitivity and specificity (*p*<0.05) for CIN2+ between the observations, but not in accuracy. In a low resource setting, specificity is important to reduce colposcopy workload. In a high resource setting like Norway, sensitivity is more important to reduce the number of cervical cancer. Specificity of cytology can be improved by HPV test in a triage of ASC-US / LSIL. The costs of a high number of HPV tests are of minor importance in a high resource setting. In the USA, co-testing (cytology and HPV test) every five years is recommended for women 30–60 years of age [10, 14].

## Conclusions

Cervical cancer screening based on cytology has limited accuracy. The study revealed a moderate agreement between the observers, along with a trade-off between sensitivity and specificity. This might indicate that hospitals with high detection rate of cervical cytology have higher sensitivity for CIN2+, but lower specificity.

## Additional file

Additional file 1: Table S1. Diagnoses per pathologist (P2–P5) in samples with Normal cytology at UNN. **Table S2.** Diagnoses per pathologist (P2–P5) in samples with ASC-US cytology at UNN. **Table S3.** Diagnoses per pathologist (P2–P5) in samples with LSIL cytology at UNN. **Table S4.** Diagnoses per pathologist (P2–P5) in samples with ASC-H cytology at UNN. **Table S5.** Diagnoses per pathologist in samples with HSIL cytology at UNN. **Figure S1.** Distribution of high-grade cytology (ASC-H+) diagnoses by observer (P2–P5) in women with at least one high-grade cytology (*N*=61). **Figure S2.** Distribution of high-grade cytology (ASC-H+) diagnoses by observer (P2–P5) in women with histological CIN2+ in follow-up (*N*=32). (PDF 100 kb)

## Abbreviations

ASC-H: Atypical squamous cells – cannot exclude HSIL
ASC-US: Atypical squamous cells of undetermined significance
CIN: Cervical intraepithelial neoplasia, also known as cervical dysplasia
CIN1, CIN2, CIN3: Cervical intraepithelial neoplasia grade 1, 2 or 3, also known as low-grade, moderate or severe cervical dysplasia
CIN2+, CIN2, CIN3: Adenocarcinoma in situ (ACIS) or cervical cancer
DNA: Deoxyribonucleic acid
HPV DNA test: Cobas 4800 detects DNA from 14 high-risk HPV types (16, 18, 31, 33, 35, 39, 45, 51, 52, 56, 58, 59, 66 and 68) at clinically relevant infection levels
HPV: Human papillomavirus
HSIL: High-grade squamous intraepithelial lesion
LBC: Liquid-based cytology
LSIL: Low-grade squamous intraepithelial lesion
NPV: Negative predictive value
Pap smear: the Papanicolaou test, also known as Pap test, cervical smear or cervical cytology
PPV: Positive predictive value
RNA: Ribonucleic acid
WHO: The World Health Organization.
