# Supplementary material for: Accuracy of cervical cytology: comparison of diagnoses of 100 Pap smears read by four pathologists at three hospitals in Norway
Source: BMC Clin Pathol. 2017 Aug 29;17:18. doi: 10.1186/s12907-017-0058-8 (PMC5576325; doi:10.1186/s12907-017-0058-8)

Supplemental

Table S1. Diagnoses per pathologist (P2–P5) in samples with Normal cytology at UNN

Patient	P2	P3	P4	P5	HPV	Biopsy
1	0	0	0	0	na	na
2	1	0	0	0	na	na
3	1	1	0	0	na	na
4	0	0	0	0	na	na
5	0	0	0	0	na	na
6	3	1	1	0	na	na
7	1	0	0	0	na	na
8	3	1	0	0	na	na
9	0	0	0	0	Neg	na
10	1	1	1	0	na	na
11	0	0	0	0	na	na
12	0	0	0	0	na	na
13	0	0	0	0	na	na
14	0	0	0	0	na	na
15	1	0	0	0	na	na
16	0	0	0	0	na	na
17	0	0	0	0	na	na
18	0	0	0	0	na	na
19	0	0	0	0	na	na
20	0	0	0	0	na	na

0 = Normal
1 = ASC-US
2 = LSIL
3 = ASC-H
4 = HSIL

na = not available

Neg = HPV DNA negative (Cobas 4800)

Table S2. Diagnoses per pathologist (P2–P5) in samples with ASC-US cytology at UNN

Patient	P2	P3	P4	P5	HPV	Biopsy
21	3	1	0	0	POS	CIN2
22	0	0	0	0	POS	na
23	1	1	1	0	POS	na
24	3	1	1	0	Neg	CIN1
25	2	2	1	0	Neg	na
26	1	1	0	0	Neg	na
27	1	0	0	0	Neg	na
28	3	3	1	4	Neg	na
29	1	1	1	1	Neg	na
30	3	1	0	0	POS	na
31	3	0	1	0	na	na
32	0	4	0	4	POS	na
33	1	1	0	0	Neg	na
34	1	2	0	0	POS	CIN1
35	1	3	0	1	Neg	Normal
36	3	1	0	0	POS	na
37	3	0	0	0	POS	CIN1
38	1	4	1	4	Neg	Normal
39	1	2	0	1	Neg	na
40	3	4	1	1	Neg	na

0 = Normal
1 = ASC-US
2 = LSIL
3 = ASC-H
4 = HSIL

na = not available

Neg = HPV DNA negative (Cobas 4800)

POS = HPV DNA positive (Cobas 4800)

CIN1 = cervical intraepithelial neoplasia grade 1 (low grade)

CIN2 = cervical intraepithelial neoplasia grade 2 (high grade)

Table S3. Diagnoses per pathologist (P2–P5) in samples with LSIL cytology at UNN

Patient	P2	P3	P4	P5	HPV	Biopsy
41	2	3	2	2	POS	Normal
42	2	2	2	2	POS	CIN1
43	4	1	1	1	Neg	Normal
44	1	2	0	3	POS	CIN1
45	2	2	2	1	Neg	na
46	4	4	3	1	POS	CIN2
47	2	2	2	2	POS	Normal
48	2	2	2	2	Neg	na
49	2	2	1	1	POS	CIN1
50	3	4	0	1	POS	CIN1
51	3	3	1	0	POS	CIN2
52	3	1	1	3	POS	CIN1
53	2	2	1	2	na	na
54	2	3	0	1	POS	CIN1
55	1	0	1	2	POS	Normal
56	1	0	1	1	POS	CIN1
57	1	1	0	2	POS	CIN2
58	3	3	0	3	Neg	Normal
59	2	2	1	2	POS	CIN1
60	2	1	1	0	POS	CIN1

0 = Normal
1 = ASC-US
2 = LSIL
3 = ASC-H
4 = HSIL

na = not available

Neg = HPV DNA negative (Cobas 4800)

POS = HPV DNA positive (Cobas 4800)

CIN1 = cervical intraepithelial neoplasia grade 1 (low grade)

CIN2 = cervical intraepithelial neoplasia grade 2 (high grade)

Table S4. Diagnoses per pathologist (P2–P5) in samples with ASC-H cytology at UNN

Patient	P2	P3	P4	P5	HPV	Biopsy
61	2	2	2	4	Neg	Normal
62	2	3	0	0	POS	Normal
63	2	4	2	3	POS	CIN1
64	2	1	1	3	POS	CIN1
65	3	2	1	4	POS	CIN1
66	4	4	1	4	na	CIN3
67	4	4	0	1	na	CIN2
68	3	4	0	1	POS	CIN1
69	1	2	2	1	POS	CIN1
70	4	3	3	4	na	CIN1
71	4	4	3	0	POS	CIN3
72	3	4	3	1	POS	CIN2
73	4	2	3	3	POS	CIN2
74	4	0	0	0	POS	Normal
75	4	4	2	2	POS	CIN3
76	4	2	3	4	POS	CIN1
77	4	3	4	4	POS	CIN2
78	4	1	4	4	POS	CIN3
79	4	4	4	4	POS	CIN3
80	4	2	3	4	POS	CIN2

0 = Normal
1 = ASC-US
2 = LSIL
3 = ASC-H
4 = HSIL

na = not available

Neg = HPV DNA negative (Cobas 4800)

POS = HPV DNA positive (Cobas 4800)

CIN1 = cervical intraepithelial neoplasia grade 1 (low grade)

CIN2 = cervical intraepithelial neoplasia grade 2 (high grade)

CIN3 = cervical intraepithelial neoplasia grade 3 (high grade)

Table S5. Diagnoses per pathologist in samples with HSIL cytology at UNN

Patient	P2	P3	P4	P5	HPV	Biopsy
81	4	4	3	4	na	CIN3
82	2	2	2	3	POS	CIN2
83	4	3	3	4	na	CIN2
84	4	4	4	4	POS	CIN3
85	4	4	1	3	na	CIN2
86	4	3	4	1	Neg	CIN2
87	4	4	4	4	POS	CIN3
88	4	4	4	4	POS	CIN3
89	4	3	4	4	POS	CIN2
90	3	2	2	3	POS	CIN1
91	4	4	4	4	POS	CIN2
92	4	2	3	4	POS	CIN2
93	4	3	4	4	POS	CIN2
94	4	4	4	4	POS	CIN2
95	4	4	4	4	POS	CIN3
96	4	3	4	3	POS	CIN2
97	4	4	4	4	na	CIN3
98	4	4	4	4	na	CIN3
99	4	1	1	1	POS	SCC
100	4	4	4	3	na	CIN1

0 = Normal
1 = ASC-US
2 = LSIL
3 = ASC-H
4 = HSIL

na = not available

Neg = HPV DNA negative (Cobas 4800)

POS = HPV DNA positive (Cobas 4800)

CIN1 = cervical intraepithelial neoplasia grade 1 (low grade)

CIN2 = cervical intraepithelial neoplasia grade 2 (high grade)

CIN3 = cervical intraepithelial neoplasia grade 3 (high grade)

SCC = squamous cell carcinoma (cervical cancer)

Figure S1. Distribution of high-grade cytology (ASC-H+) diagnoses by observer (P2–P5) in women with at least one high-grade cytology (N=61)

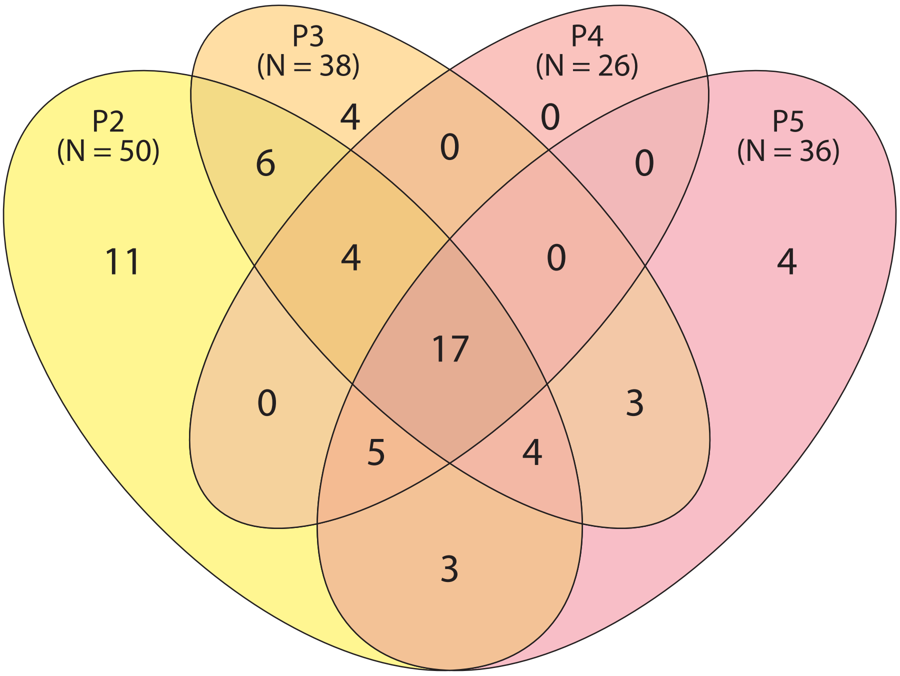

Figure S2. Distribution of high-grade cytology (ASC-H+) diagnoses by observer (P2–P5) in women with histological CIN2+ in follow-up (N=32)

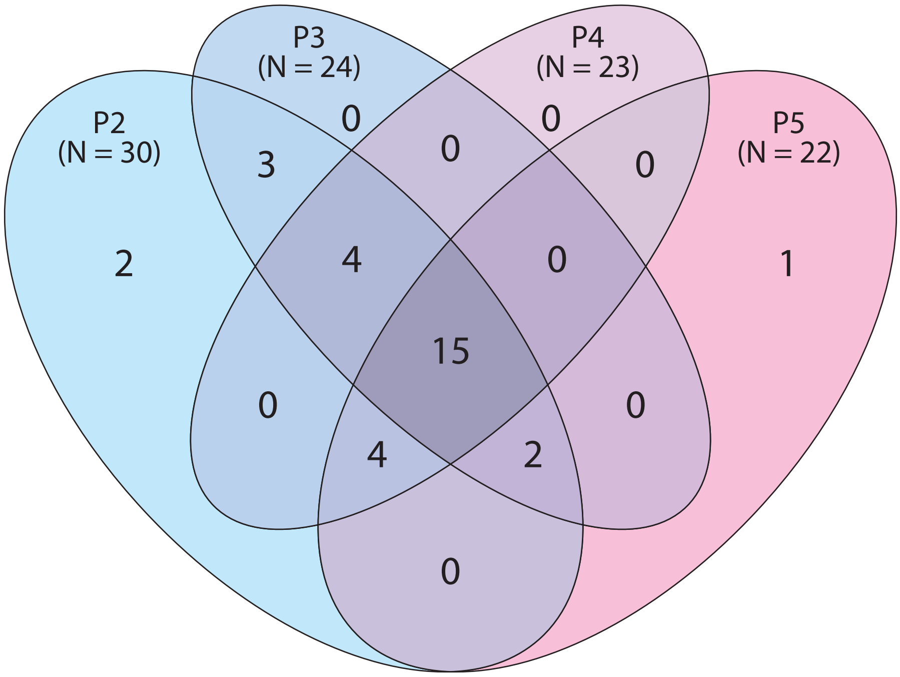

Supplement: Additional file 1: Table S1. — Diagnoses per pathologist (P2–P5) in samples with Normal cytology at UNN. Table S2. Diagnoses per pathologist (P2–P5) in samples with ASC-US cytology at UNN. Table S3. Diagnoses per pathologist (P2–P5) in samples with LSIL cytology at UNN. Table S4. Diagnoses per pathologist (P2–P5) in samples with ASC-H cytology at UNN. Table S5. Diagnoses per pathologist in samples with HSIL cytology at UNN. Figure S1. Distribution of high-grade cytology (ASC-H+) diagnoses by observer (P2–P5) in women with at least one high-grade cytology (N=61). Figure S2. Distribution of high-grade cytology (ASC-H+) diagnoses by observer (P2–P5) in women with histological CIN2+ in follow-up (N=32). (PDF 100 kb) [file 12907_2017_58_MOESM1_ESM.pdf]
